# The Gut-Brain-Axis on the Manifestation of Depressive Symptoms in Epilepsy: An Evidence-Driven Hypothesis

**DOI:** 10.3389/fphar.2020.00465

**Published:** 2020-04-08

**Authors:** Mohd. Farooq Shaikh, Chooi Yeng Lee, Win Ning Chen, Faiz Ahmed Shaikh

**Affiliations:** ^1^Neuropharmacology Research Strength, Jeffrey Cheah School of Medicine and Health Sciences, Monash University Malaysia, Bandar Sunway, Malaysia; ^2^Global Asia in 21^st^ Century (GA21) Multidisciplinary Platform, Monash University Malaysia, Bandar Sunway, Malaysia; ^3^Tropical Medicine & Biology Multidisciplinary Platform (TMB), Monash University Malaysia, Bandar Sunway, Malaysia; ^4^School of Pharmacy, Monash University Malaysia, Bandar Sunway, Malaysia; ^5^School of Pharmacy, Management and Science University, Shah Alam, Malaysia

**Keywords:** epilepsy, depression, gut-brain-axis, gut microbiome, vagus nerve

## Abstract

Epilepsy is a severe neurological disorder involving 70 million people around the globe. Epilepsy-related neuropsychiatric comorbidities such as depression, which is the most common, is an additional factor that negatively impacts the living quality of epilepsy patients. There are many theories and complexities associated with both epilepsy and associated comorbidities, one of which is the gut-brain-axis influence. The gut microbiome is hypothesized to be linked with many neurological disorders; however, little conclusive evidence is available in this area. Thus, highlighting the role will create interest in researchers to conduct detailed research in comprehending the influence of gut-brain-axis in the manifestation of depressive symptoms in epilepsy. The hypothesis which is explored in this review is that the gut-brain-axis do play an important role in the genesis of epilepsy and associated depression. The correction of this dysbiosis might be beneficial in treating both epilepsy and related depression. This hypothesis is illustrated through extensive literature discussion, proposed experimental models, and its applicability in the field. There is indirect evidence which revealed some specific bacterial strains that might cause depression in epilepsy.

## Introduction

Epilepsy is a disease of the central nervous system (CNS) which incurs episodes of epileptic seizures, accompanied by psychiatric consequences. In 2005, the Task Force of the International League Against Epilepsy (ILAE) expressed both theoretical definitions of “seizures” and “epilepsy” and the operational definitions which were used for clinical diagnosis. For a theoretical definition, epilepsy is characterized by epileptic seizures, with cognitive and neurobiological consequences. An epileptic seizure is an event of abnormal brain neuronal activity. In contrast, the operational or clinical definition requires the following situations: 1) At least two reflex seizures occurring more than 24 h apart 2) One unprovoked seizure and a probability of further seizures (≥60%) after two unprovoked seizures, occurring over the next ten years and 3) have been diagnosed with an epilepsy syndrome (Fisher et al., 2014).

The prevalence of this complex illness affects nearly 70 million people worldwide in a variety of forms and severity. In advanced countries, the incidence is around 40–70 per 100,000/year, whereas in less advanced countries, it is higher, approximately 100–190 per 100,000/year (Sander, 2003). Epilepsy (idiopathic or primary) incidence is prominent in early childhood and after adolescence in developed countries (Stephen and Brodie, 2000) while, incidence is higher in developing countries of the childhood group (Klein et al., 2003; Olafsson et al., 2005). Epilepsy, both incidence and prevalence, is observed to be elevated in males than females in both developed and developing countries. This could arguably be due to the presence of female sex hormones, estrogen, and progesterone, which may affect the threshold of seizure to some extent (Lavados et al., 1992). Few studies are available about the prevalence of epilepsy based on race and ethnicity. Prevalence of epilepsy is high in African-Americans (8.2/1,000) and non-South Asians (7.8/1,000) as compared to Caucasians (5.4/1,000) and South Asians (3.6/1,000) (Wright et al., 2000; Banerjee et al., 2009). However, it is not conclusive as it can be subjected to the lack of control of the socioeconomic status between the groups, posing a limitation to the study.

The etiology of epilepsy includes a variety of CNS disorders from parasitic infections. A common parasitic infection which affects the intracranial region is neurocysticercosis, from pork tapeworm larva, *Taenia solium* infection. It is a frequent parasitosis of the CNS with seizures as a typical manifestation (Medina et al., 1990). It was revealed that the proportion of neurocysticercosis in people with active epilepsy displayed consistency, estimated to be 29% from several studies performed in endemic areas of Latin America, Sub-Saharan Africa, and Southeast Asia (Ndimubanzi et al., 2010; Nash, 2014). This suggests the significance of neurocysticercosis as an etiology of epilepsy. Other microbial infections include bacterial such as tuberculous meningitis, whereby epilepsy manifests in the form of intracranial tuberculomas (Bahemuka and Murungi, 1989).

Another etiology of epilepsy for adults is head or brain injuries leading to cranial trauma whereas, in children, it is mainly febrile convulsions which are acute neurological disturbances. Genetic risk factors are also involved. Individuals with a first-degree epileptic relative have a threefold risk of developing one too (Senanayake and Roman, 1993). Seizure syndromes may arise from ion-channel disorders, progressive myoclonus epilepsies, neurogenetic disorders from developmental abnormality, energy metabolism defects or metabolic disturbances, as well as neuronal migration disorders (Steinlein, 2008). Lastly, environmental causes of epilepsy can be contributed by potential neurotoxic agents such as benzene hexachloride pesticide, utilized as a food grain preservative in the Lakhimpur Kheri district of India (Khare et al., 1977). There are several other seizurogenic chemicals such as the organophosphorus (OP) nerve agent and dichlorodiphenyltrichloroethane (DDT) which disrupts the modulation of sodium ion channels by delaying the action potential falling phase, inducing the intermittent release of nerve impulse. This causes tremors and seizures (Jett, 2012).

The gut microbiome is hypothesized to be linked with many neurological disorders; however, little conclusive evidence is available in this area. Thus, highlighting the role will create interest in researchers to conduct detailed research in comprehending the influence of gut-brain-axis in the manifestation of depressive symptoms in epilepsy. The hypothesis which is explored in this review is that the gut-brain-axis do play an important role in the genesis of epilepsy and associated depression. The correction of this dysbiosis might be beneficial in treating both epilepsy and related depression.

### Depression in Epilepsy

Among the many psychiatric disorders documented, depression has prevailed to be the most common comorbidity in epileptic patients (Grover, 2017). Depression is associated with loss of mood/interest, anhedonia, appetite or weight alterations, disrupted sleep and psychomotor activity, repetitive thoughts of death, and reduced energy and fatigue that persist. Despite epilepsy being a risk for depression, a bi-directional relationship is suggested whereby patients with clinical depression history is observed to have a higher likelihood of epilepsy, about four to six times (Kanner, 2003). Depressive disorders in epilepsy patients can generally be classified into three types—major depressive disorder (MDD), dysthymic disorder, and depressive disorder, which includes minor depression (Runeson and Rich, 1994). MDD and dysthymic disorder differ in terms of severity, persistence, and chronicity, but share common symptoms as mentioned above. MDD is diagnosed when there are recurring major depressive episodes for at least 2 weeks, whereas dysthymic disorder is a chronic disorder with persisting symptoms for ≥ 2 years. Minor depression is suggested if patients have less than five symptoms (Moore and Brown, 2012).

The active prevalence of depression in epileptic patients is not precise due to variations in study settings, demographic differences, and debatable methodologies employed to detect depression or epilepsy. However, the prevalence was found to be 23.1% for active depression in patients with epilepsy (Fiest et al., 2013). The lifetime prevalence of depression in epileptic patients is approximately 6% and 30% in population-based studies, and as high as 55% observed in patients of tertiary centers (Kanner, 2003; Gnanavel, 2017). Hence, the risk of depressive disorders is elevated by twofold in epilepsy compared to the standard population. The prevalence of depressive symptoms was assessed in epileptic Nigerians using the Beck Depression Inventory (BDI) for quantitative assessment and Hamilton Rating Scale for Depression (HRSD) was carried out. It was revealed the frequency of depressive symptoms was 42% and 45% respectively. There was a crucial difference between patients and controls on both scales and higher depression scores in women compared to men (Ogunrin and Obiabo, 2010).

The link between depression and epilepsy is significantly more profound when compared to other chronic medical conditions. A study examined the depression prevalence in epileptic patients as compared to patients with asthmatic conditions or healthy control individuals delineated that depressive symptoms are common in epileptic people (36.5%) as opposed to asthmatic people (27.8%) and healthy controls (Ettinger et al., 2004). The presence of depressive symptoms could be related to recurrent seizures in epileptic patients. Additionally, depression occurs in only 4% of patients free from seizures, approximately 10% in those with rare occasions of seizure attacks (less than once a month), whereas these percentages are elevated to 21% in patients having more seizure attacks and uncontrollable epilepsy. Consistent findings further strengthen the relationship whereby epileptic patients and persistent seizures have a predisposition to experience depression compared to patients in remission (Jacoby et al., 1996; O'Donoghue et al., 1999).

The bi-directional relationship, although not causal, between depression and epileptic patients could suggest common pathogenesis in both states, whereby one disorder may potentially expedite the development of another. The neurobiology perspective of depressive epileptic patients involves common pathogenetic mechanisms which could be abnormal activity or levels of neurotransmitters such as serotonin, noradrenaline, dopamine, GABA, and glutamate. The study involving genetic epilepsy-prone rats found that the serotonergic and noradrenergic pre- and postsynaptic transmission deficit rats exhibited severe seizures. When compared to the rats, MDD patients displayed similar abnormalities in the endocrine system (Jobe et al., 1995). Other studies investigated the effect of pharmacologic reduction in serotonin on seizures in humans. It was found that monoamines depletion such as serotonin has increased rate and gravity of seizures in epileptic patients (Kanner, 2005). Other potential contributing factors include the abnormalities in certain neuroanatomic regions, based on its structure and function, in depression and epileptic seizure, commonly associating with comorbid depression.

Based on neuroimaging, studies, in primary MDD, there exist alterations in the various neuroanatomic structures morphologically and volumetrically, especially frontal atrophy which includes medial prefrontal cortex, frontal cortex, and dorsolateral prefrontal cortex atrophy (Zhao et al., 2014; Bludau et al., 2016). Meta-analyses have also recorded a decrease in volume of the cingulate cortex found in MDD patients compared to healthy individuals (Rodriguez-Cano et al., 2014) and depressive symptoms cause hippocampal atrophy (Buddeke et al., 2017). It was documented that patients with temporal lobe epilepsy have frontal lobe dysfunction. This dysfunction leads to a reduction in inferofrontal metabolism and interferes serotonergic neuron transmission. Hence, it is speculated that frontal lobe dysfunction can predispose the patient to depression (Menzel et al., 1998). Studies have also revealed the prevalence rate of depression ranges from 19% to 65% among epileptic patients of mesial temporal or frontal lobe origin (Kanner and Balabanov, 2002). Overall, depression in epilepsy may arise from neurotransmitter dysfunction and neuroanatomic changes.

### Current Treatments for Depression in Epilepsy

With the high prevalence and the burden of the disease, which resulted in the mortality of depression manifested in suicide, many patients often go undiagnosed and without proper treatment. In a clinical assessment of 174 patients with chronic epilepsy, MDD was a common diagnosis when they were interviewed using the Mini-International Neuropsychiatric Interview (MINI) and mood disorders modules. It was also found less than half were given antidepressant medications as a treatment (Jones et al., 2005).

Frequently prescribed anti-depressants to epileptic patients are from classes such as the selective serotonin reuptake inhibitors (SSRIs) and serotonin-noradrenaline reuptake inhibitors (SNRIs). They act as primary drugs or treatment for depression due to greater tolerability and decreased adverse effects as compared to classic tricyclic antidepressants (TCAs) which have been known to worsen seizure activity and decreasing seizure threshold in a dose-dependent manner (Dailey and Naritoku, 1996; Elger et al., 2017). The mechanism of action for both inhibitors blocks different transporters at the presynaptic neuronal membrane and elevate serotonin and/or noradrenaline levels in the synapse (Cardamone et al., 2013). Citalopram treatment for four consecutive months at 20 mg per day for depression in epilepsy patients reduced the total number of seizures from 1.32 to 0.82 seizures/month, and 67% of patients in the study experienced considerable improvement or remission (18%) of depressive symptoms (Specchio et al., 2004).

The pro-convulsant effect of venlafaxine (SNRI) was absent, and as the dose increased from 20 to 40 mg/kg/d, it did not significantly lower seizure threshold (Ahern et al., 2006). However, venlafaxine overdose can induce seizures, but at therapeutic dosage, it is safe to be used (Pisani et al., 2002). Available literature suggests that long-term SSRI/SNRI anti-depressant treatment does not exacerbate seizure frequencies in patients and even some experience complete freedom from seizure activity during the anti-depressant treatment. There were only a few exceptional cases reported whereby individuals did experience worse seizure frequencies which could be reversed by removal of anti-depressant or increasing anti-epileptic medications (Specchio et al., 2004; Okazaki et al., 2011). It was also recorded the anti-depressants effects on seizure activity from approximately 75,000 epileptic patients which revealed a significant reduction in seizure incidence of depressed patients on anti-depressants as compared to placebo-treated, suggesting a potent anti-seizure effect when given at therapeutic doses (Alper et al., 2007).

There exist another class of medication known as the noradrenergic and specific serotonergic anti-depressants (NaSSAs) which are psychiatric drugs used primarily as anti-depressants. In the NaSSAs class, mirtazapine was studied for its risks of epileptic events in adults (20–64 years) while on this anti-depressant. A follow-up of 5 years was conducted and it was found that mirtazapine, sertraline, and escitalopram did not heighten the risk of seizure activity out of the 11 drugs examined (Hill et al., 2015). Similar evidence was observed in another large study which utilized the drug safety data information. It was concluded that SSRIs and the mirtazapine anti-depressants might pose as a better candidate to treat depression in patients with enhanced seizure risk as compared to tricyclic anti-depressants (Koster et al., 2013).

Nevertheless, there exist some limitations with employing anti-depressants into the medication regime of an epileptic patient. There could be a pro-convulsant potential of the anti-depressants as mentioned earlier, which often leads to lowering the seizure threshold and thus exacerbating seizure activity. The seizure risk associated with anti-depressant administration also varies between the type of drugs whereby maprotiline and amoxapine has a greater risk compared to doxepin, at smaller chances. However, the SSRI class is believed to have a reduced risk of inducing seizures (Pisani et al., 1999; Habibi et al., 2016). Another limitation includes the potential pharmacokinetic interactions between the anti-epileptic and anti-depressant medication *via* the cytochrome P-450 pathway involved in drug metabolisms. Anti-epileptic medications, including phenytoin and barbiturates, can induce P-450 isoenzymes (CYP1A2 and CYP3A4) which depresses the level of the antidepressant medications. In contrast, SSRIs such as sertraline and fluoxetine inhibit isoenzyme metabolism and elevate anti-epileptic blood levels (Trivedi and Kurian, 2007).

The common mode of actions anti-depressants share is their modulation of biological chemicals in the body, such as serotonin and norepinephrine. The beneficial effect of administering anti-depressants can be reaped by initiating the treatment in patients with epilepsy with careful surveillance of plasma drug levels of both types of medications, anti-epileptic, and depressant as well as for any pharmacokinetic interactions. The selection of antidepressants with the slightest interaction may be appropriate to develop a pharmacological tool to treat depressive symptoms in epileptic patients (**Table 1**).

**Table 1:** Type of anti-depressant drugs and their effect on seizures.

Drug	Class	Mode of action	Effect on seizures
Imipramine Desipramine Amoxapine Doxepin	TCAs	Inhibit the uptake of norepinephrine and serotonin in adrenergic and serotonergic neurons	Lower the seizure threshold
Sertraline Fluoxetine Citalopram	SSRIs	Blocks the SERT which induces selective inhibition of serotonin uptake at the presynaptic neuronal membrane	Helps in seizures control
Desvenlafaxine Duloxetine Venlafaxine	SNRIs	Blocks the NET	Decrease in seizure frequency
Mirtazapine	NaSSAs	Antagonizing the adrenergic α2-autoreceptors and α2-heteroreceptors, blocks 5-HT_2_ and 5-HT_3_ receptors	Safe in epilepsy

TCAs, tricyclic antidepressants; SSRIs, selective serotonin reuptake inhibitors; SERT, serotonin reuptake transporter; SNRIs, serotonin-noradrenaline reuptake inhibitors; NET, noradrenaline transporter; NaSSAs, noradrenergic and specific serotonergic anti-depressants.

### Depression in Epilepsy and the Gut-Brain-Axis

**Figure 1:**
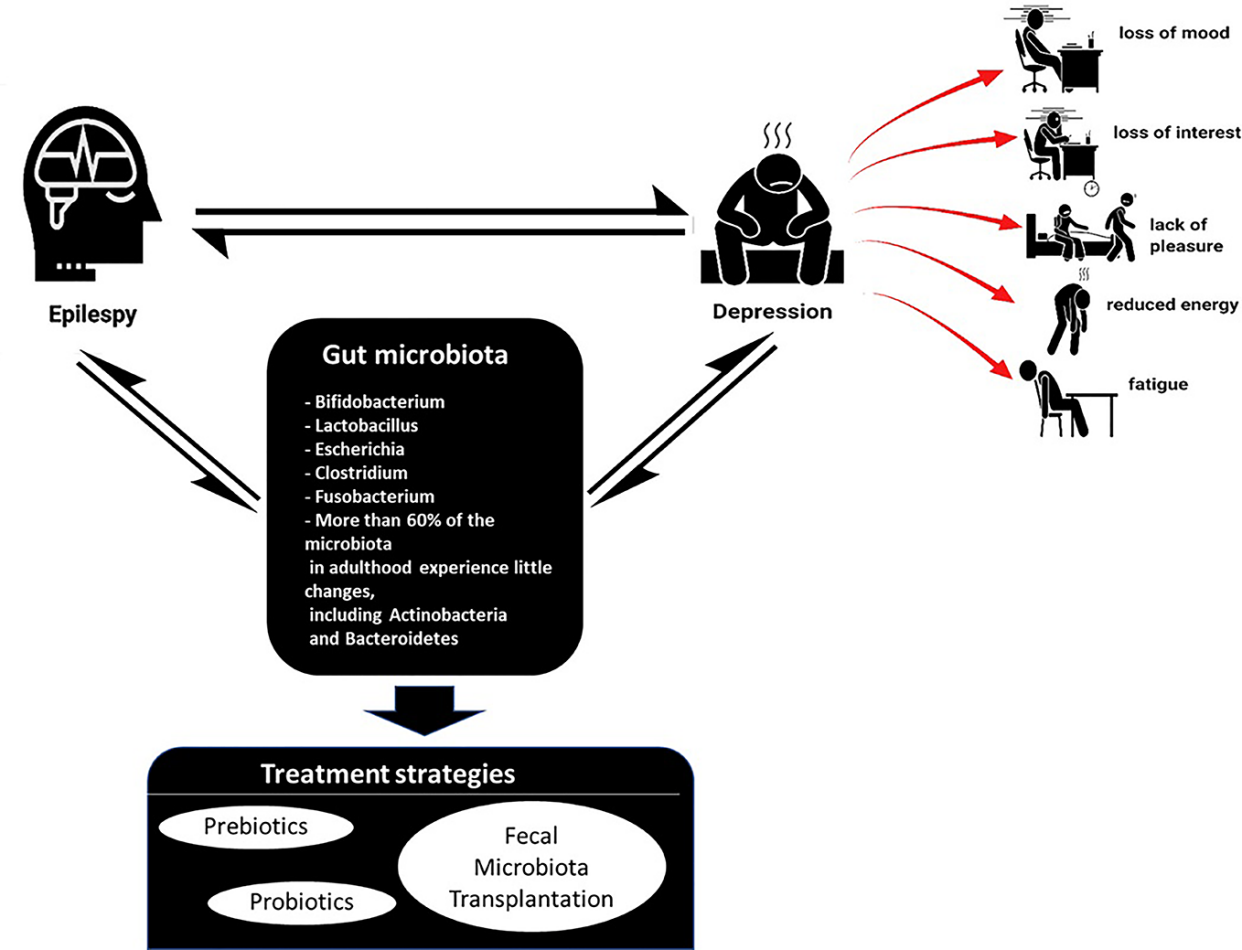
Commonly found gut microbiome in epilepsy and depression & possible treatment strategies.

Depression and epilepsy may no longer be considered diseases of the CNS only. They appear more complex than that. Depression is also a co-morbidity of several other disorders such as obesity, irritable bowel syndrome (IBS), chronic fatigue syndrome, and type-2 diabetes mellitus (Slyepchenko et al., 2017). Emerging shreds of evidence have suggested a potential role of the gut microbiota in the pathophysiology of depression (Jiang et al., 2015; Zheng et al., 2016). However, there is limited literature which reviews the link between depression to gut microbiota by different groups of researchers (Slyepchenko et al., 2017; Patist et al., 2018; Schachter et al., 2018) (**Figure 1**).

The type of food an individual consumes will have its influences on the gut and its immune system. A high-fat diet (HFD) changes and interacts with the microbiota composition, activates intestinal mast cells, and elevates the secretion of pro-inflammatory cytokines from the macrophages (C, 2013). The intestinal microbiota may play a part impacting cognition, mood, anxiety, and depression, as well as CNS physiology. The microbial imbalance or maladaptation, also known as dysbiosis, have been suggested to correlate with various neurodegenerative disorders (Paoli et al., 2014; Jiang et al., 2017; Parashar and Udayabanu, 2017), autism (Evangeliou et al., 2003), depression (Murphy et al., 2004), multiple sclerosis (Storoni and Plant, 2015), seizure susceptibility in epilepsy (Dahlin and Prast-Nielsen, 2019), and cancer (Scheck et al., 2012; Allen et al., 2014).

### Gut Microbiota-Depression

The promising effects of probiotics in depression were reported in few studies. Rats induced to have depression had decreased brain noradrenaline levels, and increased peripheral IL-6 secretion and amygdala corticotrophin-releasing factor mRNA levels. However, probiotic *Bifidobacterium infantis* treatment reversed the behavioral deficits, reinstate basal noradrenaline concentration, and normalized immune response (Desbonnet et al., 2010). A different probiotic strain *Lactobacillus rhamnosus* modulated the GABAergic system of normal, healthy mice (Bravo et al., 2011). The *L. rhamnosus* reduced GABA_B1b_ mRNA expression in the hippocampus, amygdala, and *locus coeruleus* of the mice suggesting an antidepressant-like effect of GABA_B_ receptor antagonists (Slattery et al., 2005). *L. rhamnosus* also altered the levels of hippocampal expression of GABA_Aα1_ and GABA_Aα2_ mRNAs, which may have contributed to the antidepressant-like behavior of the animals. The effects of *L. rhamnosus* were abolished in vagotomized mice, reinforcing the contribution of the vagus nerve in relating the gut and the brain (Bravo et al., 2011). Germ-free (GF) mice exposed to forced swimming test have decreased depression-like behavior compared with specific pathogen-free mice. However, GF mice with microbiota originating from MDD patients, have elevated depression-like behavior compared to GF mice with microbiota derived from normal, healthy individuals. The microbiota of MDD patients was characterized by considerable changes in *Firmicutes*, *Actinobacteria*, and *Bacteroidetes* abundance (Zheng et al., 2016). Analysis of fecal microbiota of MDD patients by other groups of researchers also showed changes in a relative abundance of *Bacteroidetes* although the shift in microbial compositions regarding healthy controls differed across studies (Naseribafrouei et al., 2014; Jiang et al., 2015).

Several labs have shown that anxiety-, depression-like behavior, and cognitive impairment occurring from gastrointestinal disease, a high-fat diet or antibiotic usage linked with abnormalities in the gut microbiota. However, supplementation by specific *Lactobacillus fermentum* NS9 strain restored and regulated the microbiome of the gut (Hu et al., 2015; Wang et al., 2015). The likelihood of depressive symptoms rooted in abnormal gut microbiota is further explored, whereby it was found that depressed rats possessed different microbiota as compared to control rats. Citalopram was given and it alleviated the defects but did not restore the microbiota, whereas *Lactobacillus helveticus* NS8 treatment improved both situations (Liang et al., 2015), suggesting the pivotal role of commensal microbiota in mental disorders.

### Gut Microbiota-Epilepsy

There is evidence which suggests a relationship of gut microbiota in epilepsy. A case-report involving a 22-year-old Crohn's disease patient received fecal microbiota transplantation (FMT) treatment and displayed seizure-free events despite discontinuation of antiepileptic drug with sodium valproate during the 20 months follow-up (He et al., 2017). It was also revealed that probiotic treatment decrease seizure frequencies (at least 50%) in 28.9% of the patients with drug-resistant epilepsy (Gomez-Eguilaz et al., 2018). Moreover, a study illustrated the differences or the alterations of gut microbiome composition in patients with drug-resistant epilepsy (n = 42) and patients with drug-sensitive epilepsy (n = 49) whereby in drug-resistant patients, there were more abundant rare bacteria from the phylum *Firmicutes* as compared to the latter. Through this study, it was also found that *Bifidobacteria* and *Lactobacilli* incur less than four seizure events per year (Peng et al., 2018).

Additionally, a diet that has shown positive effects on neurological disorders and neurodegenerative diseases is known as the ketogenic diet (KD). Of note, KD is an established therapy alternative for therapy-resistant epilepsy treatment or medically refractory epilepsy in children only. KD is a high in fat with adequate protein intake, and low-carbohydrate diet, at the ratio of fat to protein and carbohydrates, 3:1. Hence, approximately 70–90% of the energy intake will be obtained from fat. Treatment with KD resulted in a more than 50% reduction in the seizure in about half of the treated children (Freeman et al., 1998; Neal et al., 2009). The mode of action behind the KD is the diet induces ketosis. Subsequently, the ketones are utilized as a substitute substrate for cellular ATP production (Dahlin and Prast-Nielsen, 2019). The metabolic shift incurs biochemical, hormonal, and metabolic changes which may cause a decrease in neuronal excitation and seizures events. However, ketosis may not be solely responsible for seizure response and there could exist several other hypotheses of the modes of action in KD which could play a more prominent role.

Through this diet, it may affect gut health. It was recently found that the relative abundance of the beneficial gut bacteria, bifidobacteria*, E. rectale*, and *Dialister* decreases while *E. coli* increases in a therapy-resistant epileptic patient after KD diet for 3 months (Lindefeldt et al., 2019). Prebiotics can restore the number of bifidobacteria, while reducing *E. coli* and enterococci in human trials (Turroni et al., 2016) and probiotics reduced the number of seizures by more than 50% of epileptic patients receiving probiotics as a complementary treatment (Gomez-Eguilaz et al., 2018). Therefore, there is a need to understand the effect of KD-induced changes in gut microbiota on the therapeutic effects of KD, the influence of KD on the overall gut health, and if concurrent intake of prebiotics or probiotics is required during a KD treatment.

### Gut Microbiota-Depression in Epilepsy

Gut microbiota alteration has distinctively shown to be parallel with the pathophysiology of depression and epilepsy. From present evidence, the link between microbiota profile in MDD and epilepsy remains speculative. However, there is indirect evidence drawn from other studies that may provide insight into common, specific bacteria strain indicative of depression in epilepsy. As mentioned earlier, MDD is a co-morbidity of IBS. Importantly, IBS elevates epilepsy risk (Chen et al., 2015). Similar to MDD, IBS patients have increased gut permeability (Zhou et al., 2009; Gecse et al., 2012), resulting in an influx of LPS (Dlugosz et al., 2015), and elevated pro-inflammatory cytokines (Liebregts et al., 2007; An et al., 2016). The alterations of the fecal microbiome under the influence of KD in children with epilepsy were investigated. After 3 months on KD intervention, it was revealed that the relative abundance of *Bifidobacterium* was considerably lowered in patients through whole metagenomic sequencing, suggesting the role of the microbiota in seizure susceptibility and the potential anti-seizure efficacy in KD treatment (Lindefeldt et al., 2019).

IBS patients who also have MDD are less responsive to psychotherapy and antidepressants (Whorwell et al., 1987; Drossman et al., 2000), hence maintaining the makeup of gut microbiota through prebiotics and probiotics appears to be an ideal treatment for depression in epilepsy. In support of this notion, research has indicated that depression, anxiety, and panic attacks (Schnorr and Bachner, 2016; Liang et al., 2018a) may be from abnormal gut microbiota. The robust link between depressive symptoms to the gut microbiota can be seen in research conducted whereby the GF or microbiota-depleted animals presented defective brain development and abnormal mental growth (Luczynski et al., 2016; Chen et al., 2017), indicating that both neuroplasticity and myelin plasticity is regulated by the gut microbiota. In light of this, regulation or rectifying the microbiota abnormalities could alleviate the disorder by improving the microbiota-gut-brain axis and growth of the brain and behavior. Hence, the remedial or beneficial effects from microbiota regulation or therapies targeting the microbiota have continuously gained popularity through attempted treatments by microbiota intervention using probiotics, prebiotics, and fecal microbial transplantation.

Overall, the possible link between the gut microbiota and depression in epilepsy has enhanced the research interest in the gut microbiota as a therapeutic agent. The gut microbiota plays an important role in regulating the host's cognitive functions, mood, and emotion through the microbiota-gut-brain axis, consisting of three pathways 1) nerve, 2) neuroendocrine, and 3) immune pathways (Liang et al., 2018b). The gut microbiota is able to regulate neurotransmitter synthesis *via* modification in neurotransmitter-based metabolism pathways and possibly impacting the expression of neurotransmitter-related genes. Different bacteria strains synthesize different neurotransmitters such as Bacillus and few lactic acid bacteria (LAB) strains, synthesize acetylcholine and catecholamines (Wall et al., 2014) whereas Candida, Streptococcus, Escherichia, and Enterococcus can synthesize 5-HT (Holzer and Farzi, 2014), which makes up 90% of the body's 5-HT (Margolis et al., 2014). Various coryneform and LAB strains make glutamate (Glu), and synthesize GABA (Mazzoli and Pessione, 2016). The neuroactive property of gut microbiota regulation could be further exploited as a potential therapy or treatment in depression in epilepsy as the root of both depression and epilepsy is speculated to originate from dysfunction in neurochemistry.

### Alternative Treatment Therapies for Depression in Epilepsy

Alternative treatments also include various psychotherapies, pharmacotherapies, and their combinations. The efficacy of psychotherapies in depression was reported to be possibly better than anti-depressants in decreasing relapse (Thompson et al., 2010; Walker et al., 2010). It was also shown that stimulation of vagal nerve was efficient for treatment-resistant epilepsy and depressive patients with sustained effects over time by elevating brain-derived neurotrophic factor (BDNF) expression in the rat brain by phosphorylation of TrkB (Furmaga et al., 2012). However, the combination of drug- and therapy-based treatment such as vagus nerve stimulation needs to be carefully considered. It has been demonstrated by a mouse model study whereby it employed a combination of SSRIs pharmacotherapy, fluoxetine, and fear-extinction training. This combination eliminated conditioned fear as compared to individual treatment which was ineffective and did not elicit any beneficial response (Karpova et al., 2011).

The supplementation of probiotics as adjuvant therapy for alleviating symptoms of depression in epilepsy may be proposed to manage the disorder better. As depression in epilepsy encompasses is a heterogeneous disorder, manipulation of the gut microbiota may offer a similarly effective treatment as compared to drug-based medication. Despite the immediate occurrence of the physiological effects in most antidepressants, the therapeutic effect is only apparent after continued usage for weeks and in some patients (15–30%), the associated side effects may discontinue its usage (Gartlehner et al., 2005).

Lately, a new term has been coined, encephalobiotics, consisting of probiotics, prebiotics, postbiotics, microbes, microbial parts, or agents that could manipulate the microbiota for improvement of cognition (Prescott and Logan, 2016). In contrast, psychobiotics are live bacteria (probiotics) that confer remedial mental benefits upon ingestion in patients suffering from psychiatric illnesses by interacting with the commensal gut bacteria (Sarkar et al., 2016). By applying these therapies to alleviate the symptoms of depression in epilepsy, it could indeed eliminate certain barriers for effective treatment. However, the mechanism of action of these therapies as a treatment for depression in epilepsy needs to be further elucidated. One may propose the mode of action involves the regulation of inflammatory markers and neurotransmission (Wallace and Milev, 2017). It would be worthwhile to investigate the connection between the central and enteric nervous systems as they may work in tandem to produce the beneficial effects of biotics supplementation. More future studies including more in-depth animal and human studies alike, are needed before these therapies can be regarded as a front-line treatment for alleviating the symptoms of depression in epilepsy.

Despite the new strategies, the process of selecting the type of therapy and its suitability to the patient is complex as it takes into account many playing factors such as the specific syndromes experienced by the patient, records of the illness and its corresponding treatment, physical comorbidities, risk of inducing drug interactions, and lastly, patient compliance toward the treatment. Moreover, it is imperative to provide considerable evidence regarding any additive and synergy of combined therapies and medication for depression in epilepsy treatment. Epileptic patients who experience emotional and mental deterioration must be recognized promptly and diagnosed accordingly. This is possible through dependable screening instruments and executing the appropriate medical support or referral to a professional such as a psychiatrist. Overall, the present paper has analyzed the hypothesis which proposed the influence of gut microbiota in depression in epilepsy. Detailed studies are needed for conclusive evidence to understand further the role and the manipulation of the gut microbiota in the gut-brain axis to increase its applicability and relevance in treating patients with depression in epilepsy using alternative treatment strategies.

## Author Contributions

MS and CL conceptualized the idea. MS, CL, WC, and FS had contributed in the literature review and manuscript writing. MS and WC have revised and edited the manuscript.

## Conflict of Interest

The authors declare that the research was conducted in the absence of any commercial or financial relationships that could be construed as a potential conflict of interest.
